# Charge densities in actinide compounds: strategies for data reduction and model building

**DOI:** 10.1107/S2052252519010248

**Published:** 2019-08-07

**Authors:** Christopher G. Gianopoulos, Vladimir V. Zhurov, A. Alan Pinkerton

**Affiliations:** a Department of Chemistry and Biochemistry, University of Toledo, Toledo, OH 43606, USA

**Keywords:** charge densities, augmented multipole refinement, QTAIM, molecular crystals, actinides, uranium

## Abstract

The data quality requirements for charge density studies on actinide compounds are extreme. Important steps in data collection and reduction required to obtain such data are summarized and evaluated. The steps involved in building an augmented Hansen–Coppens multipole model for an actinide pseudo-atom are provided. The number and choice of radial functions, in particular the definition of the core, valence and pseudo-valence terms are discussed. The conclusions in this paper are based on a re-examination and improvement of a previously reported study on [PPh_4_][UF_6_]. Topological analysis of the total electron density shows remarkable agreement between experiment and theory; however, there are significant differences in the Laplacian distribution close to the uranium atoms which may be due to the effective core potential employed for the theoretical calculations.

## Abstract 2

A three part paper on charge density analysis of actinide containing compounds is presented that covers experimental protocols, augmented multipole model building and a comparison of topological analysis of experimental and theoretical electron densities.

## 1. Introduction   

The nature of *f*-element bonding is poorly understood and presents a challenge from both experimental and theoretical perspectives (Küchle *et al.*, 1994 ▸; Prodan *et al.*, 2007 ▸; Kaltsoyannis, 2013 ▸; Kaltsoyannis & Kerridge, 2014 ▸), especially for the early to middle actinides where relativistic effects cannot be neglected (Pyykkö, 1988 ▸; Onoe *et al.*, 1993 ▸). Participation of the 5*f* or 6*d* orbitals (or both) in covalent bonding has been considered for many years, although the amount of covalent character in the highly polar actinide-element bond is still a subject of debate. In contrast to the chemically familiar orbital overlap concept of covalency in light elements, covalency in actinides may be enhanced by small energy differences between metal and ligand orbitals (Su *et al.*, 2018 ▸; Ingram *et al.*, 2008 ▸; Tanti *et al.*, 2018 ▸; Gregson *et al.*, 2016 ▸; Kerridge, 2017 ▸; Kaltsoyannis, 2013 ▸, 2016 ▸, 2018 ▸; Jones *et al.*, 2013 ▸; Kirker & Kaltsoyannis, 2011 ▸; Prodan *et al.*, 2007 ▸; Neidig *et al.*, 2013 ▸; Kelley *et al.*, 2017 ▸). This observation has provoked further debate as to whether ‘genuine’ covalency ought to increase or decrease across the actinide series. Further complicating matters, experiment and theory indicate that the filled 6*s* and 6*p* orbitals also respond to bonding in actinide-element compounds, and are commonly described as pseudo-core orbitals (Boring *et al.*, 1974 ▸; Onoe *et al.*, 1993 ▸; Pyykkö, 1988 ▸; Neidig *et al.*, 2013 ▸; Denning, 2007 ▸). Experimental insight into *f*-element bonding has predominately been obtained spectroscopically, for example, by means of UV–Vis (absorption and fluorescence) (Jorgensen *et al.*, 1963 ▸), photoelectron (Dau *et al.*, 2012 ▸), Mössbauer (Kalvius, 1986 ▸) and, more recently, X-ray absorption spectroscopy (Minasian *et al.*, 2012 ▸; Jollet *et al.*, 1997 ▸), although such measurements involve a convolution of the ground- and excited-states. Charge-density studies and topological analysis of the total electron density in the ground state provide a unique opportunity to study different bonding interactions simultaneously and result in quantitative characterization of the bonds present in a crystal, and the combined experimental and theoretical approach provides a more comprehensive understanding of such kinds of interactions. Accurate charge-density experiments are often in remarkable agreement with theoretical predictions in the case of typical light-atom structures and are well represented in the literature (Gianopoulos *et al.*, 2016 ▸; Chua *et al.*, 2017*b* ▸,*a* ▸; Tidey *et al.*, 2017 ▸). Such studies were regarded as ‘almost impossible on heavy-atom systems’ (Lander *et al.*, 1986 ▸) only 30 years ago, but since then the area of experimental X-ray charge-density investigations has greatly expanded and improved, benefitting from considerable advances in computing, diffraction instrumentation and experimental methodologies. In our opinion, these improvements have made charge-density studies on heavy-atom systems an attractive opportunity for further development of the field, although such experiments are still extremely challenging. Thus, recent work in our laboratory concerning Cs_2_UO_2_Cl_4_ (Zhurov *et al.*, 2011*a* ▸,*b* ▸) and [PPh_4_][UF_6_] (Gianopoulos *et al.*, 2017*b* ▸) has demonstrated that accurate charge densities of uranium complexes can be obtained from an in-house X-ray diffraction system at low temperatures (20 K). The adequacy of these results can be confirmed by topological analysis of the resulting total ground-state electron density and by comparison with results derived from theoretical charge densities in the context of Bader’s Quantum Theory of Atoms in Molecules (QTAIM) (Bader, 1994 ▸). Experimentally determined bond descriptors are in fair agreement with those obtained from high-level theoretical calculations (Tanti *et al.*, 2018 ▸; Gianopoulos *et al.*, 2017*b* ▸; Wellington *et al.*, 2016 ▸), although theoretical results may show a dependence on the methods applied (Schreckenbach & Shamov, 2010 ▸).

Our goal herein is to demonstrate that experimental charge-density studies of heavy-atom systems are worthwhile challenges and provide opportunities to refine our understanding of actinide-element bonding and inform theoretical developments in the area. The following covers results from three areas of our approach presented as one cohesive story. In short, we will describe (i) improvements in our data collection and reduction strategy, (ii) modifications to the traditional Hansen–Coppens multipolar formalism to increase its flexibility when applied to actinide elements and (iii) the outcome of these efforts for [PPh_4_][UF_6_]. In this context, the following will relate our observations and opinions developed over the last several years, as well as difficulties arising well before the stage of model building. Some of these concerns, while well known, still merit discussion and require careful correction to the primary data. Such effects include application of a flood field adjustment, corrections for strong absorption, harmonic contamination of monochromated X-rays (Gianopoulos *et al.*, 2017*a* ▸; Kirschbaum *et al.*, 1997 ▸), oblique incidence correction, multiple scattering and resolution-dependent radiation damage to the sample. Although heavy-atom systems are significantly more complicated when compared with light-atom structures, we believe that combined theoretical and experimental charge-density studies provide attractive opportunities for understanding the nature of actinide-element bonding.

## 2. Collection and treatment of primary data   

Although the experimental details and methods applied for the processing of the primary data have been previously described, in the following, we have reprocessed the original diffraction data obtained for [PPh_4_][UF_6_] at 20 K (Gianopoulos *et al.*, 2017*b* ▸), and tested different multipole refinement protocols using both *XD2006* (Volkov *et al.*, 2006 ▸) and *MoPro* (Jelsch *et al.*, 2005 ▸). We have thus obtained a significantly improved result, as well as informing the charge density community on potential protocols to follow and pitfalls to avoid. Thus we will expand on what we have found to be important considerations for obtaining charge-density quality data sets for heavy-atom containing systems, keeping in mind that the quality of those data must be significantly higher than for a light-atom charge density analysis.

**Figure 1:**
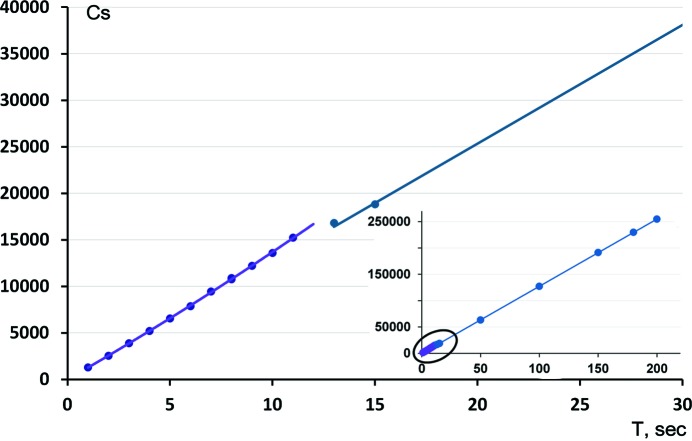
Calibration curve for the response of PMT1 and PMT2. The full calibration curve is shown in the inset.

To begin with, we prefer to use a 0.5 mm-wide collimator which provides no less than 95% of the maximum beam intensity for the 0.3 mm area at the crystal position rather than a sharply focused one (Zhurov *et al.*, 2008 ▸). This removes the need for a correction for inhomogeneous illumination of the sample. Carefully optimized data integration, which takes into account α_1_/α_2_ splitting, is essential. Developed for this purpose, the program *VIIPP* (Zhurov *et al.*, 2008 ▸, 1999 ▸) with an image plate floodfield correction, ability to linearize detector response, background and reflection profiles averaged over the whole data set, and rejection of partial or overlapped reflections provides superior data. Careful alignment of the detector system eliminates a significant number of possible errors. For example, the Rapid II detector utilizes separate photomultiplier tubes (PMTs) for strong (PMT2) and weak data (PMT1). We have found that the signal-to-noise ratio can be increased by optimizing the tube voltage for PMT1 (usually by decreasing it), followed by appropriate adjustment of the tube voltage for PMT2. Reduced measured intensities in the case of decreasing the PMT1 voltage can be compensated for by increasing exposure time. For data accuracy, the gain in signal-to-noise for weak data is more important than the decrease of measured intensities. We also have found that non-linearity of the PMTs response should be taken into account. In our case, we have measured the intensity of the direct beam with attenuators at several exposure times providing about 1000 counts s^−1^ at the beam center. A slight non-linearity was observed for PMT1 (Fig. 1 ▸); whereas the PMT2 response appeared to be linear within the accuracy of the measurements. We have also observed that over time the response of the PMTs is subject to slight drifting. Periodic recalibration of our PMTs is helping to ensure that the highest quality data are obtained.

**Figure 2:**
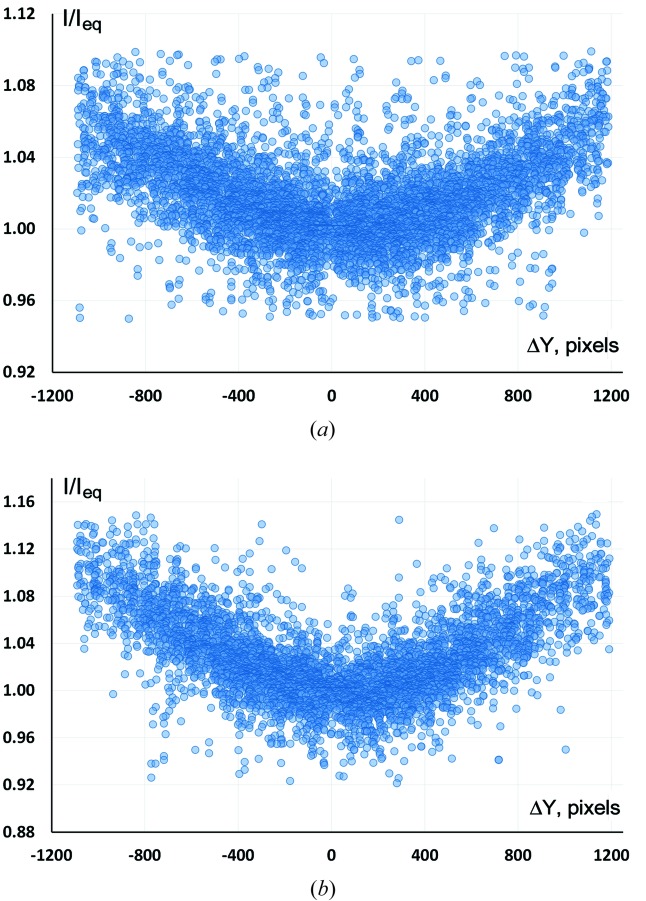
Oblique incidence effect for RAXIS RAPID detector with (*a*) Mo *K*α and (*b*) Ag *K*α radiaton, determined from a spherical ruby crystal and plotted as a function of vertical distance from the equatorial plane in pixels, based on intensity ratios of equivalents to the one within ±100 pixels from the equatorial plane.

In addition to the PMT recalibration, we have also found that an oblique incidence effect (Wu *et al.*, 2002 ▸; Zaleski *et al.*, 1998 ▸), although small for the image plate used in RAPID detectors, still has to be accounted for. We have measured the value of this correction by equivalents comparison using a spherical ruby crystal. The magnitude of the deviation varies with source wavelength and its behavior is shown in Fig. 2 ▸ for Mo *K*α and Ag *K*α radiation. The correction has been incorporated into our integration software *VIIPP*. The effect is smaller for the less penetrating Mo *K*α radiation (deviation at IP edges is up to ∼6% on average), *R*_merge_ still improves from ∼2.0 to ∼1.5% for the spherical ruby test crystal. For Ag *K*α, the improvement in *R*_merge_ is doubled, from ∼2.5 to ∼1.5%.

There are several sources which contribute to over-estimation of weak data, such as multiple diffraction, contamination with cosmic radiation and electronic noise spikes. Application of *I*/σ(*I*)-based cutoff prior to averaging also elevates weak data. For this reason, we use all measured equivalents including negative intensities and only apply an *I*/σ(*I*) cutoff after merging. High data redundancy lowers statistical counting errors but it is often difficult to obtain redundancy as high as desired for the highest-order observations, which are inherently weaker. Plots of |*F*_obs_|/|*F*_calc_| for high-angle reflections demonstrate that the errors associated with weak high-order data essentially average out; however, we find that inclusion of data with *I* < 3σ(*I*) introduces undesirable noise in the experimental electron density, as judged on the basis of Fourier difference maps. Including data with *I* < 3σ(*I*) would be justifiable in the case of zero ‘average’ noise, unfortunately, in our system – due to the aforementioned reasons – this average is positive and produces bias in the refined scale factor, where slight errors in the scale factor are known to introduce bias in the charge-density model (Becker, 1977 ▸; Rees, 1978 ▸; Stevens & Coppens, 1975 ▸). Despite rejecting some unique data due to applying the sigma cut, the data-to-parameter ratio is still very high owing to the high resolution of the measurement 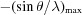 = 1.3 Å^−1^ (Mo) or 1.7 Å^−1^ (Ag).

The use of helium cooling (20 K) provides significant improvement to data quality by removing thermal diffuse scattering, increasing intensities and accuracy of high-order data, and reducing the amplitude of both harmonic and anharmonic atomic displacements more prevalent at higher temperatures (Larsen, 1995 ▸). Proper separation of thermal motion from the aspherical electron density is necessary to obtain a meaningful multipole model. While the boost in scattering power is considerable at helium temperatures relative to nitrogen cooling, the added benefit to the reduction of thermal motion due to lower temperature cannot be ignored. Moreover, the increased scattering power, particularly for high-angle data, allows for shorter frame times and experiments, which enables the measurement of a highly redundant data set fairly quickly (1–2 days) using a large area detector (Zhurov *et al.*, 2008 ▸).

Crystal size and shape are also extremely important. A small (∼100 µm) isotropically shaped crystal would be ideal in order to minimize the effects of anisotropic absorption and extinction, while still having enough scattering power to keep frame lengths reasonable. In the case of [PPh_4_][UF_6_], the crystal was a prism of dimensions 0.17 × 0.14 × 0.09 mm.

A careful absorption correction is necessary for heavy-atom systems and to that end we have obtained our best results using a numerical absorption correction as previously described and implemented in the program *CCDABS* (Zhurov & Tanaka, 2003 ▸). Briefly, this method involves photographing the crystal with a high-resolution digital camera. Photographs are taken at several degree increments about the φ axis (typically 5°), providing 72 or more still images of the crystal covering a complete 360° rotation. The direct space outer contour of the crystal is then determined from pairs of mirrored images at the positions φ and φ + 180°. The outlining contours at these positions will therefore be identical, other than mirrored. Both images are used simultaneously for the boundary determination at angles of φ and φ + 180° to improve accuracy as it may be difficult to identify from only one image of the pair due to shadows and glare at certain crystal positions. From the obtained set of contours, we then reconstruct a series of limiting planes which determine the crystal shape. The recovered three-dimensional shape is then used for accurate numerical absorption correction. This method has proven to be extremely useful for crystals of arbitrary shape or when faces cannot be clearly seen, making it troublesome to apply absorption corrections based on face-indexing. For the example of [PPh_4_][UF_6_] and prior to other corrections described below, *R*_merge_ improved from 3.23 to 2.87% when comparing an empirical method, as implemented in the program *SORTAV* (Blessing, 1995 ▸, 1997 ▸), with numerical absorption correction performed with *CCDABS*.

Following scaling and merging of the absorption corrected data, several additional corrections may be necessary including corrections for harmonic contamination of the ‘monochromatic’ X-ray beam (Gianopoulos *et al.*, 2017*a* ▸; Kirschbaum *et al.*, 1997 ▸) and radiation damage to the sample. The presence of harmonic contamination is a machine-specific property. All crystals with small absorption will be affected identically. In the case of strongly absorbing crystals, a secondary effect will also be observed due to the difference of the absorption and anomalous scattering coefficients at λ and different harmonics of λ (Gianopoulos *et al.*, 2017*a* ▸). Although the correction for harmonic contamination is generally small, it is especially important for weak low-angle reflections, *e.g.* see the case of triaminotrinitrobenzene, wherein inclusion of the over-estimated, uncorrected 001 and 003 reflections in the original data set were responsible for unreasonable polarization of the electron density perpendicular to the molecular plane (Chua *et al.*, 2017*a* ▸).

**Figure 3:**
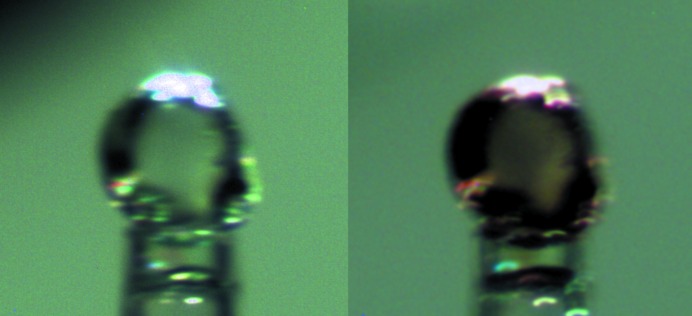
Crystal of [NBnEt_3_]_2_[UCl_6_] before and after 24 h of data collection at 20 K.

Based on our experiences to date, many crystals of uranium compounds are prone to radiation damage even at 20 K, and the [PPh_4_][UF_6_] system is no exception. In some cases where the radiation damage is severe, it is worthwhile to re-measure with another crystal, as we have noticed that different crystals from the same sample tend to decay at different rates depending on crystal quality. Radiation damage causes weakening of intensities which increases with resolution, the effect being similar to an increase of thermal motion. Diagnosis of severe radiation damage may be obvious, for example, color change (see Fig. 3 ▸), but can also be diagnosed by comparison of the scaling of low-resolution and high-resolution data separately. If radiation damage is present, the scaling factors of the high-resolution data will increase considerably with frame number, but for the low resolution they will only increase slightly. A traditional way of dealing with radiation damage is introducing an overall *B*-factor for each frame in addition to the scale factor; this has proven useful in macromolecular crystallography where radiation damage is a common problem (Lomb *et al.*, 2011 ▸). These values can be determined by refinement during the scaling and merging process or by calculating the ratios of the scaling factors for the high- and low-resolution data, assuming that the distribution of reflections participating in scaling is similar for each frame.

The accuracy of high-order data influences the magnitude of high-frequency noise in charge density maps and therefore how well the features near nuclear positions are resolved, especially for very heavy atoms, owing to their high charge density compared with light atoms. The decay may affect intensities of high-resolution data by an order of magnitude. In this case, the reconstruction of the undisturbed electron density is very complicated. Strongly decaying systems are thus likely to be poor candidates for charge-density studies with heavy atoms, and indeed we have abandoned several compounds/datasets as radiation damage was too severe. Typically, we try to work with crystals that exhibit narrow, well formed and symmetrical diffraction peaks. This is generally observed for high-quality crystals which we have found to be less prone to decay than those of lower quality. Unfortunately, the magnitude of decay in many cases can only be estimated after data are collected.

Another significant source of error in measured intensities is the presence of multiple diffraction (MD). In the case of area detectors with limited degrees of goniostat freedom, the MD effect is unavoidable. While such effects were first described between 1920 and 1940 by Berg (1926 ▸), Wagner (1923 ▸) and Renninger (1937 ▸), contamination of primary data by MD is rarely accounted for (Zhurova *et al.*, 1999 ▸; Tanaka *et al.*, 1994 ▸; Sakakura *et al.*, 2014 ▸). However, as noted by Speakman and others, MD ought not to be ignored when extremely accurate data are required (Speakman, 1965 ▸), *i.e.* for charge-density studies and especially for heavy-atom containing systems (Tanaka *et al.*, 1994 ▸). Multiple diffraction events can attenuate or amplify the intensity of a measured reflection, known as *aufhellung* and *umweganregung*, respectively. In general, MD contaminates strong and weak reflections in different ways, namely, the important MD events attenuate the intensity (*aufhellung*) of strong data while increasing the intensity (*umweganregung*) of weak reflections (Tanaka *et al.*, 1994 ▸). In the case of a four-circle diffractometer equipped with a point detector, a protocol to avoid the collection of strongly contaminated data can be developed (Tanaka *et al.*, 1994 ▸). However, implementation of such a routine for imaging plate data is a considerable challenge.

**Figure 4:**
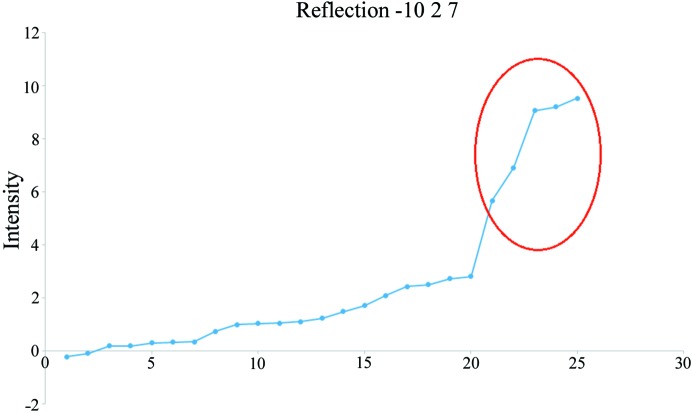
Reflections −10 2 7 in [PPh_4_][UCl_6_] sorted by intensity showing contamination by the *umweganregung* MD effect (five strongest measurements, outlined in red, rejected in this extreme case).

In order to analyze the contamination of measured intensities, we carefully examined images and equivalent reflections measured with sufficient redundancy, as previously suggested (Macchi *et al.*, 2015 ▸; Otwinowski & Minor, 1997 ▸). A simple graphical tool visualizing the distribution of equivalents for each independent reflection, sorted by intensity (Fig. 4 ▸), can be very useful for identifying outliers. Manual inspection of the data, though time consuming, allows for more confident outlier rejection from the dataset. In general, equivalents rejected in such a manner amount to about 1% of the total measured reflections. Even though the improvement of *R*_merge_ is slight (a few hundredths of a percent), the decrease in the noise level of residual density maps is noticeable. In the case of [PPh_4_][UF_6_], decay correction improved *R*_merge_ by ∼1% after absorption correction, followed by manual rejection of 417 out of 75 976 (0.55%) measured intensities, which reduced *R*_merge_ by ∼0.08% to yield a final *R*_merge_ of 1.68% for all data.

As a rule of thumb, we try to work with a dataset that has *R*_merge_ < 1.75%. Small differences in *R*_merge_ may not appear significant, but datasets with *R*_merge_ > 2% are noisier and more challenging to model to the point that we would likely abandon charge-density modeling on a dataset with *R*_merge_ > 3%. While the incremental improvements described above have proven to be necessary for our heavy elements program, we note that our small-molecule datasets have also benefitted from the careful attention to improving our methodology. For example, we recently obtained *R*_1_ = 0.68% for a multipole refinement on the energetic small-molecule salt TKX–50 at 20 K [monoclinic *P*2_1_/*c*; (sinθ/λ)_max_ = 1.33 Å^−1^; data/parameter = 12.34; Tidey *et al.*, 2017 ▸].

## 3. Modifications of the traditional Hansen–Coppens formalism   

**Figure 5:**
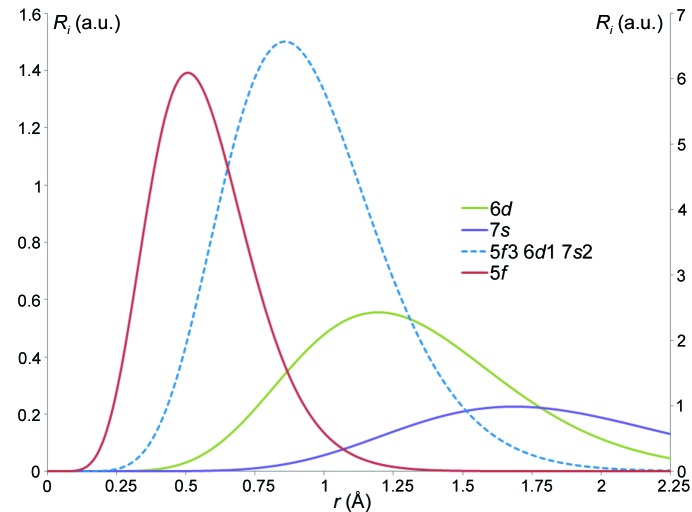
Non-normalized, one-electron single-ζ radial functions for the U atom for the valence levels 7*s*, 6*d* and 5*f*, depicting each separately as well as the weighted average corresponding to the 5*f* ^3^ + 6*d* ^1^ + 7*s* ^2^ configuration. The more contracted 5*f* radial distribution is plotted according to the right axis, whereas the other more diffuse radial functions are plotted on the left axis.

In the Hansen–Coppens multipolar formalism, the electron density of a crystal is modeled as the sum of pseudo-atoms, which traditionally employ three parts [equation (1)][Disp-formula fd1], namely (i) a spherical frozen core, (ii) a spherical valence part with *P*_v_ and κ_*s*_ parameters describing the valence charge and expansion/contraction of this term, and (iii) the aspherical portion of the valence density represented as a sum of multipolar terms with the appropriate expansion/contraction coefficients (κ*_l_*). 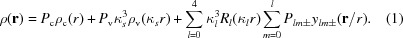
Generally, the difference between such a representation and a spherical atom model reflects a small but chemically meaningful perturbation. In the case of light-atom structures, the traditional multipolar formalism has been extremely successful and can accurately recover total electron densities based on the excellent agreement between experimentally and theoretically derived properties. Nevertheless, while for light elements such as carbon, a single mixed radial term (*R_l_*) is sufficient to describe the hybridized 2*s*2*p* orbital; for heavy elements with very different orbitals involved in the valence shell, a single mixed radial term representing them all simultaneously is inappropriate. For example, the ground-state configuration of uranium is [Rn]5*f* ^3^6*d* ^1^7*s* ^2^, spanning three principal quantum numbers which have very different radial behavior (Fig. 5 ▸).

Moreover, there is significant overlap between the radial distribution of the valence terms and some of the core density. It is therefore unsurprising that (i) neglecting perturbation of the relevant core electron distribution when using a large frozen core and (ii) the use of a single-ζ weighted average radial function to model the aspherical portion of the electron density do not provide a satisfactory description of the experimental electron density in the case of heavy-element compounds. In our previous work (Zhurov *et al.*, 2011*a* ▸,*b* ▸), we employed an augmented multipole model, following the example described by Farrugia & Senn (2012 ▸) by including additional aspherical terms based on four to five radial functions representing 5*f*, 6*s*, 6*p* (or averaged 6*s* + 6*p*), 6*d* and 7*s* orbitals, but keeping the frozen core. We have now redone these refinements for [PPh_4_][UF_6_] using the data reduction protocol described above, as well as using a smaller frozen core and a larger set of radial functions as suggested by theoretical studies (Cao *et al.*, 2003 ▸; Odoh & Schreckenbach, 2010 ▸; Küchle *et al.*, 1994 ▸). The modified Hansen–Coppens scheme is described below in equation (2) and is similar to those previously employed for heavy-element studies (Batke & Eickerling, 2013 ▸; Gianopoulos *et al.*, 2017*b* ▸; Zhurov *et al.*, 2011*a* ▸,*b* ▸). The single weighted-average ‘valence’ term has been replaced by a summation over several split pseudo-atoms. The parts of the complete atom are one frozen-core + ‘valence’ component and several no-core ‘valence’ only pseudo-atoms. This results in a smaller frozen core and the sum of *N* pseudo-atoms which accordingly model the spherical valence and aspherical portions of the heavy-atom electron density. We also note that the multipolar expansion has been expanded to the sixth-order, which is necessary to accurately describe *f* orbitals. 
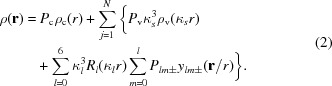
Note that the index *j* = 1…*N* has been omitted from the part of equation (2) in brackets for clarity, but is actually applied to each term.

This problem is also well known among theoreticians, as all-electron calculations are exceedingly costly for very heavy elements. A popular approximate method, which decreases computational requirements, employs relativistic effective core potentials (RECPs). In the RECP method, a transferable pseudopotential for the frozen core is defined up to a certain level of *n*, and calculations proceed with the rest of the electrons (Kahn *et al.*, 1978 ▸). Results from such calculations demonstrate that outer-core electrons play a role in the chemistry of actinides and that they may be considered as ‘pseudo-valence’ or ‘semi-core’. In the case of uranium, it has been shown that, at the minimum, the closed 6*s* and 6*p* orbitals ought to be considered in the ‘valence’ space; however, significant errors may still result from excluding electrons belonging to the *n* = 5 shell. Among the most preferred RECPs for uranium is the small-core Stuttgart RECP with 60 frozen-core electrons. This method includes all electrons with *n* ≥ 5 in the ‘valence’ space, and has been shown to minimize the significant ‘frozen core errors’ obtained with the large-core Stuttgart RECP (78 frozen-core electrons) (Schreckenbach & Shamov, 2010 ▸). Therefore it is not surprising that the best agreement between the obtained augmented Hansen–Coppens experimental models and theory is observed when the uranium partitioning is defined in a manner similar to the small-core Stuttgart RECP employed in theoretical calculations.

We have thus explored multiple ways to construct the uranium pseudo-atom with the expectation that outer-core orbitals will indeed need to be included in the aspherical portion of the multipolar formalism. Unfortunately, we have not found a perfect solution despite considerable testing, but we have been able to make incremental progress towards improving crystallographic agreement factors as well as flattening the residual density around the uranium nucleus. In the following, we will describe two new models which both employ six pseudo-atom terms for uranium partitioned in two different ways (28 and 60 frozen-core electrons) to explore the possibility of observing polarization in deeper electron shells. These will be compared with our previous communication wherein we chose to model the uranium atom with five pseudo-atoms and a frozen core including up to the 5*d* level (78 frozen-core electrons). By comparison of these partitioning schemes, we hope to identify which is most preferable for understanding the electronic structure and properties of actinide containing compounds.

### 3.1. Geometry and symmetry   

**Figure 6:**
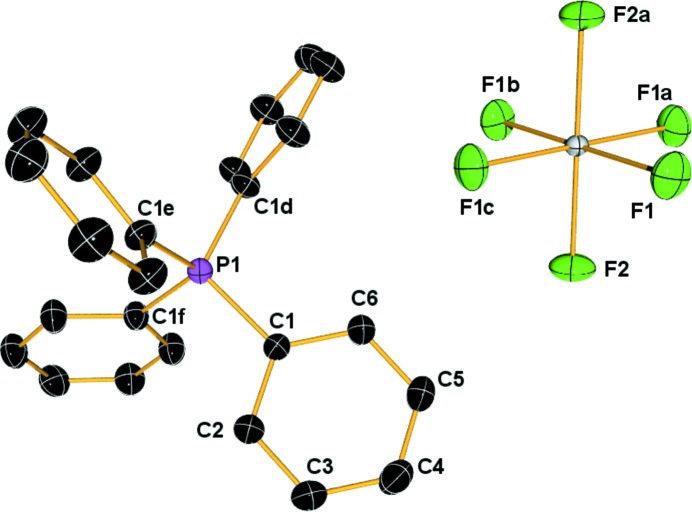
*ORTEP* plot of [PPh_­4_][UF_6_] with ellipsoids at the 99% probability level, H atoms have been omitted for clarity. Symmetry operators: a = *y*, 1 − *x*, 1 − *z*; b = 1 − *x*, 1 − *y*, *z*; c = 1 − *y*, *x*, − *z*; d = −0.5 + *y*, 0.5 − *x*, 0.5 − *z*; e = 0.5 − *y*, 0.5 + *x*, −0.5 − *z*; f = − *x*, 1 − *y*, *z*.

The compound [PPh_4_][UF_6_] crystallizes in the tetragonal space group 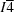, with the uranium atom located on the 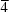 symmetry element (Fig. 6 ▸). The equatorial fluorine(s) (F1) occupy general positions and the axial F2 ligands lie on the twofold axis which is coincident with the 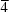 element. The phosphorus atom of the cation also lies on a position of 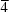 symmetry with the independent C_6_H_5_ fragment occupying general positions. The high-symmetry sites of the uranium and phosphorus atoms conveniently constrain the crystallographically allowed multipole parameters.

### 3.2. Refinement strategy   

Refinement of H, C and F atoms followed the traditional Hansen–Coppens scheme. Modeling of carbon atoms utilized a single κ_*s*_ and κ_*l*_ for all six phenyl carbons and multipole expansion up to and including *l* = 4 with no symmetry constraints imposed. All five hydrogen atoms were treated with a single κ_*s*_ and κ_*l*_ (constrained to 1.2), the multipole expansion being carried out using dipolar terms only and bond distances constrained to the average neutron values. The fluorine atoms were modeled with separate κ_*s*_ and κ_*l*_ parameters with a similarity restraint applied. All symmetry-allowed multipole parameters up to and including *l* = 4 were refined, namely all multipoles for the F1 atoms occupying a general position and those allowed by twofold symmetry for the axial F2 atoms. The modeling strategy for the phosphorus atom required some care to ultimately obtain a negative Laplacian at the critical point for the covalent P—C bond. We have chosen to split the phosphorus atom with an additional component such that the P atom is described with a frozen 1*s* ^2^ core and refined *P*_v_ and κ_*s*_ on the 2*s* ^2^ 2*p* ^6^ shell and a separate core-less component for the valence 3*s* + 3*p* electrons. With this approach, the expected negative Laplacian at the P—C bond critical point was obtained. It is worth noting that the 2*s*2*p* portion only deviates slightly from the expected spherical frozen core (<2.5% for κ_*s*_ and *P*_v_ refines to ∼7.98 e), while multipole parameters refine to ∼0 and were finally set to zero. For the 3*s*3*p* valence portion of the P atom, κ_*s*_ and κ_*l*_ were refined as well as all multipole parameters up to and including *l* = 4, allowed by the 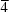 symmetry. Employment of anharmonic thermal parameters with a non-split model provided the same result as the split-core approach but utilized significantly more variables.

Following a traditional multipole refinement, the observation of high residual peaks significantly closer to the uranium nucleus than the maxima of the radial distributions of the valence electrons suggests that perturbation of the core electron density should also be accounted for (see Table 3). Therefore the inclusion of ‘semi-core’ pseudo-atoms is necessary to obtain the best models in the case of heavy-atom systems and is in agreement with the recommendation to avoid large-core pseudopotentials for theoretical calculations (Schreckenbach & Shamov, 2010 ▸). In our preferred method, the uranium atom is split into the sum of six pseudo-atoms; (i) a frozen core which contains all electrons up to the 4*f* shell and a 7*s* valence term, separate valence terms for (ii) 6*d* and (iii) 5*f*, (iv) a weighted average for 6*s* and 6*p* pseudo-valence, (v) 5*d* semi-core and (vi) a weighted average for 5*s* and 5*p*. In order to maintain the relative shell structure of the uranium atom in the collective pseudo-atoms, we have chosen to apply two separate *kappa* restraints (or constraints), *i.e.* one for the spherical *kappa* sets and one for the aspherical *kappas*, for all uranium pseudo-atoms such that the *kappa* parameters have a tight similarity restraint (or to be identical).

**Figure 7:**
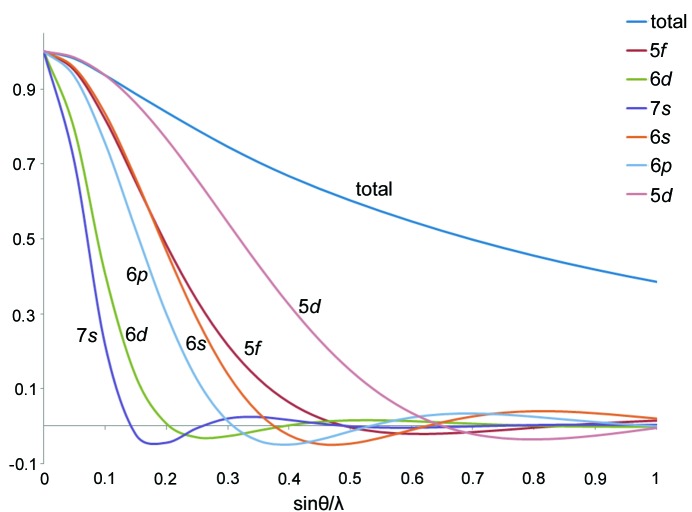
Scattering factors (normalized to one electron) for the uranium atom and selected subshells plotted against resolution up to sin(θ)/λ = 1 Å^−1^.

Refinements are started using a block refinement technique, the *P_v_* and *κ_s_* terms being refined during early block refinement stages. Generally, multipolar terms are slowly added to the uranium pseudo-atoms, as informed by inspection of the residual density, paying particular attention to the peak heights and their distance from the nucleus, and comparing with the available radial functions (Fig. 5 ▸). Avoiding correlations between the diffuse 6*d* and 7*s* terms is also a challenge and requires extremely accurate low-order data as can be seen from the scattering curves in Fig. 7 ▸. Moreover, significant correlations may appear between similar multipolar density terms, for example, in the present case, between the *z*-directed (parallel to 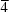) multipolar functions *P_lm_* with *m* = 0 for different *l*. Thus, while gradually adding uranium multipolar terms, we initially restrict these to a small set. For example, we first add angular terms appropriate to the orbitals anticipated to be relevant, such as *l =* 2 angular density functions for *p* pseudo-atoms, *l =* 4 for *d* orbitals and *l =* 6 for *f* orbitals. The complete multipolar expansion over the entire basis set may be added later as necessary. In the case of [PPh_4_][UF_6_], we restrict the multipolar expansion to symmetry-allowed multipoles for 4/*m* symmetry at the U pseudo-atoms, despite the crystallographic 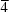 symmetry of uranium. We justify this choice on the basis that deviation from 4/*m* symmetry is only slight and inclusion of *l* = odd multipolar terms do not significantly improve the model fit, quality of difference or residual maps, or crystallographic agreement factors. That said, with multipole expansion up to and including *l* = 6 for uranium, a maximum of seven multipolar parameters can be refined per U pseudo-atom using the approximate 4/*m* symmetry compared with a maximum of eleven multipole parameters at 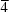 symmetry.

Despite the overlap of the tails of *n =* 4 radial density functions with the valence 5*f* orbital (Fig. S1 of the supporting information), inclusion of split pseudo-atoms for the *n =* 4 electrons does not dramatically improve refinement, although in some cases it appears to stabilize refinement of the κ*_l_* parameters. We also note that refinement of anharmonic thermal parameters (included in model **1c**, see discussion below) stabilizes the refinement of multipolar *κ_l_* parameters for the U pseudo-atoms which, in some cases, tend to drive towards being unrealistically contracted (κ*_l_* ≫ 1.5). Correlated spherical valence charges (*P*_v_) for inner shells were restrained to be equal to the number of electrons in that shell. The spherical valence charges for 5*f* and 6*d* were freely refined in both **1b** and **1c**. The charge on the 6*s* + 6*p* pseudo-atom was freely refined in the case of **1b** and refined to 7.40. Free refinement of the 6*s* + 6*p* charge was also attempted for model **1c** and provided similar results during block refinement, but refined to a population greater than eight during full refinement. Ultimately the population was restrained to its neutral value for model **1c**.

Treatment of the density due to the extremely diffuse 7*s* orbital is not straightforward. We have experimented with completely removing the 7*s* portion, using an averaged term for the diffuse 6*d* and 7*s* density functions, and have also tried to keep them separate but employ a restraint on the 7*s* valence charge. As depicted in Fig. 7 ▸, the major portion of information concerning the 7*s* density of uranium is expected to be contained in the first 16 low-order reflections (sinθ/λ < ∼0.15 Å^−1^_,_ assuming *κ_s_* and *κ_l_* = 1), and the first 36 reflections in the case of 6*d* (sinθ/λ < ∼0.20 Å^−1^_,_ assuming *κ_s_* and *κ_l_* = 1). Moreover, spatial overlap of the 7*s* radial function with the nuclear position(s) of the surrounding ligands results in strong correlation between the spherical 7*s* valence charge and the ligand *P*_v_ parameters. We concluded that the best treatment of the population of the 7*s* pseudo-atom was to restrain it to a small value which is consistent with theoretical results (Boring & Wood, 1979 ▸; Boring *et al.*, 1974 ▸; De Jong & Nieuwpoort, 1996 ▸; Hay *et al.*, 1979 ▸; Straka *et al.*, 2003 ▸).

When evaluating the quality of the model during the refinement, aside from lower *R*-factors, it is important to monitor the residual density, and normal probability and scale factor plots, as implemented in the program *DRKplot* (Stash, 2007 ▸), and the reasonableness of model parameters. In our experience, following the response observed in the scale factor plots and residual density provides suggestions for the values of well fitted model parameters. At times these may need to be frozen during certain stages of block refinement in order to stabilize the refinement of correlated parameters. Ultimately, the fact that these parameters can safely be refined at later stages suggests the adequacy of model parameters obtained by such a method. Nevertheless, the least-squares surface is typically quite flat and it is difficult to obtain stable refinements without the use of a damp parameter or chemically sensible restraints. Although this is not ideal, we are generally unable to obtain stable models without damping at this time.

### 3.3. Comparison of models   

With this general strategy we have obtained several well fitting models, see Table 1 ▸. The models presented below (**1a**, **1b** and **1c**) were designed in order to compare the effect of the uranium atom frozen core size analogous to theoretical studies, which have shown that large-core RECPs may introduce significant ‘frozen core’ (FC) errors in comparison with the small-core and all electron treatments (Odoh & Schreckenbach, 2010 ▸). Specifically, model **1a** includes 78 electrons in the frozen core, model **1b** includes 60 FC electrons and **1c** includes 28 electrons, additional details about partitioning of the aspherical portions are given in Table 1 ▸. Variations between *R*-factors, residual density maxima, scale factor plots and residual density (Table 1 ▸ and Figs. S3–S6) were taken into account when deciding which model is most preferred. For this reason we have also compared the results of topological analysis of the total electron density for each model with those from theory (Gianopoulos *et al.*, 2017*b* ▸) [B3LYP (Becke, 1993*a* ▸,*b* ▸); F: all-electron 6–31 *g*(*d*′, *p*′); U: small-core Stuttgart RSC 1997 RECP (Dolg *et al.*, 1993 ▸)].

### 3.4. Topology of the total electron density   

The reconstructed electron densities have been evaluated and compared based on the properties of the electron density and its derivatives at the (3, −1) bond critical points (bcp) following Bader’s Quantum Theory of Atoms in Molecules (Bader, 1994 ▸). Further qualitative comparisons between the models were also made by comparing maps of the deformation densities and the Laplacian of the total electron density; these will be discussed in turn below. There is fair agreement between the models’ properties and those predicted by theory (Table 2 ▸), whereas the corresponding properties obtained from a spherical atom model are quite different.

The topological properties at the U—F bcps for all three models (Table 2 ▸) demonstrate slight differences of ρ and ∇^2^ρ. Augmented Hansen–Coppens models **1b** and **1c**, which include aspherical modeling of deeper lying core electrons, are in closer agreement with our theoretical results compared with model **1a**. Moreover, previous theoretical studies have also indicated that outer-core electrons do indeed respond to perturbations resulting from metal–ligand interactions, especially in the case of 6*p* (Fryer-Kanssen & Kerridge, 2018 ▸; Denning, 2007 ▸; Bartleet *et al.*, 1992 ▸; O’Grady & Kaltsoyannis, 2002 ▸). The ‘best’ agreement between the experimental and theoretical (Gianopoulos *et al.*, 2017*b* ▸) topology is obtained with model **1b**, which has a frozen core analogous to the small-core RECP used in the theoretical model. In this case, the electron density at the bcp and its Laplacian are within 10% of the theoretical values. On the other hand, model **1c** provided the most stable refinement allowing looser restraints. In all cases, the experimental models suggest more covalent character in the U—F bonds than is predicted by theory on the basis of their QTAIM properties. Thus, when comparing bonding descriptors (Espinosa *et al.*, 2002 ▸; Gatti, 2005 ▸) of the experimental and theoretical models, for the experiment the density ρ at the bcp is higher, the Laplacian is less positive, the total electronic energy density, *h*, is more negative, the ratio of electron potential and kinetic energy densities |*v*|/*g* is larger, and the ‘covalence degree’ *h*/ρ is more negative. In the case of model **1b**, the covalence degree descriptor indicates 7% (U—F2) and 15% (U—F1) greater covalent character than theory. For all models, both experimental and theoretical, topological results place the bonding regime in the so called transit region, where bonding results from the interplay of electrostatic and covalent contributions (Espinosa *et al.*, 2002 ▸; Gatti, 2005 ▸).

**Figure 8:**
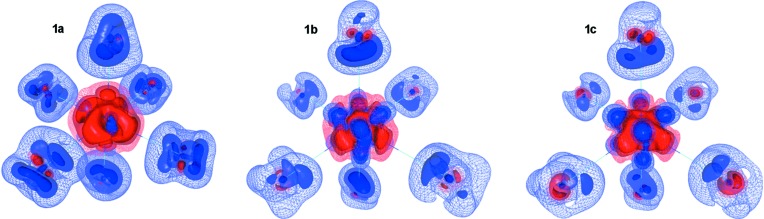
Deformation density isosurface map for the UF_6_^−^ anion obtained from models **1a**–**1c**. Charge concentrations are depicted in blue and depletions in red. The isodensity surfaces are plotted from the ±0.15 (mesh), 0.30 and 0.45 (most opaque) eÅ^−3^ levels.

Comparison of the deformation densities (DD) obtained from theory and experiment is also informative. As can be seen from Fig. 8 ▸, the deformation density around the F atoms is consistent across the models described herein. The axial F2 atoms appear to have more structure, and their DD is characterized by positive regions in the direction of the metal and also directly behind the F atom, opposite to the direction of the U—F bond. The deformation density in the direction of the metal has two maxima (see Fig. 8 ▸). A charge depletion is observed near the F2 nuclear position and extends in the direction perpendicular to the U—F bond, suggesting polarization of the electron density consistent with π-donor character in the ligand–metal interaction. The equatorial F1 atoms appear to have less structure in the deformation density maps in as much as the maximum values of the DD are not large and any maxima essentially smear together at a low isodensity level. Nevertheless, as in the case of the axial fluorine atoms, there are two maxima directed towards the metal for the equatorial F1 atoms as well.

However, the modeling strategy does affect the structure of the DD around the uranium atom. Comparing models **1a** through **1c** it can be seen that including asphericity of the outer-core electrons seems to ‘resolve’ some of the features observed in the DD. For example, the DD around the U atom (for all models) shows eight maxima at a distance of ∼0.6 Å from the U nuclear position, as expected for electron density belonging to the 5*f* and/or 6*s*—6*p* levels, and resembles what would be expected for the electron density due to a singly occupied 5*f* orbital (Kaltsoyannis & Bursten, 1995 ▸; Boring *et al.*, 1974 ▸) (FWHM for the 5*f* radial function spans from ∼0.33 to 0.74 Å whereas the FWHM for the averaged 6*s*—6*p* radial function spans from ∼0.49 to 1.02 Å, see Table 3 ▸). The ‘contrast’ between these eight concentrations and charges of other regions improves by modeling asphericity of deeper shells in the outer-core. Maximum regions of charge depletion in the DD around U are oriented along the metal–ligand bonds, as expected from ligand-field theory arguments (Fig. 8 ▸).

**Figure 9:**
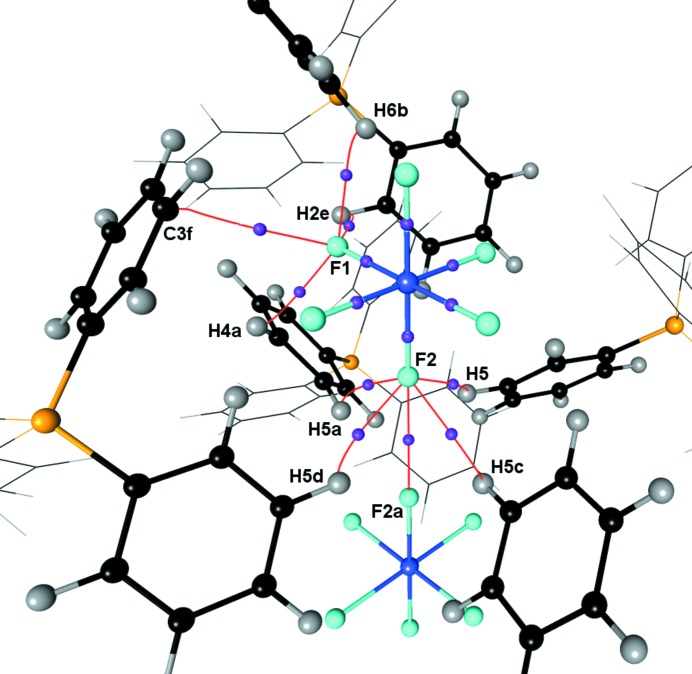
Molecular graph depicting intermolecular interactions around the independent fluorine atoms, only (3, −1) critical points are shown for clarity. F: light blue; U: blue; C: black; P: orange; H: gray; (3, −1) critical points: purple. Symmetry operators: a = *y*, 1 − *x*, 1 − *z*; b = *y*, 1 − *x*, −*z*; c = 1 − *y*, *x*, 1 − *z*; d = 1 − *z*, 1 − *y*, *z*; e = 0.5 − *x*, 1.5 − *y*, −0.5 + *z*; f = 1.5 − *y*, 0.5 + *x*, 0.5 − *z*.

It is surprising and merits further consideration that there is such a noticeable difference in the deformation density between the axial and equatorial fluorine ligands despite the fact that the tetragonal distortion is very slight (0.011 Å), although statistically significant. Under closer scrutiny, it is apparent that the crystal surroundings could be partially responsible for these differences (Fig. 9 ▸). The axial fluorine atoms participate in five intermolecular interactions for which bond paths were characterized. Of these, four involve F2⋯H interactions and the fifth involves F2⋯F2. All of the F2⋯H interactions are between F2 and H5 on four different symmetry-related cations. There are four intermolecular interactions involving F1 bonding to three different cations. These involve F1⋯H2 and F1⋯H6 on the same PPh_4_ cation, but on separate phenyl rings, as well as F1⋯H4 and F1⋯C3 on different cations. Assuming the validity of the Espinosa relationship (Espinosa *et al.*, 1999 ▸, 1998 ▸) for all such interactions, the cumulative dissociation energies of these interactions amounts to ∼23 and ∼21 kJ mol^−1^ for F2 and F1 respectively, with each F⋯H interaction contributing around 4.5–6.5 kJ mol^−1^. The dissociation energies for the F⋯C and F⋯F interactions were estimated to be less than 4 kJ mol^−1^. The QTAIM properties of the intermolecular interactions were consistent across both of the improved models **1b** and **1c** (Table S1 of the supporting information).

### 3.5. Topology of the Laplacian of the electron density   

Topological analysis of the Laplacian of the electron density, ∇^2^ρ, has been shown to reveal additional information regarding the spatial redistribution of the electron density in molecules. While the qualitative pictures obtained often resemble the charge concentrations and depletions observed in deformation density maps, the Laplacian distribution does not suffer from the requirement of a promolecule reference density. The critical points of the Laplacian distribution correspond to charge concentrations in the valence and core shells, VSCC and CSCC, respectively, and valence and core shell charge depletions, VSCD and CSCD. In the case of light-atom structures, the VSCCs correspond to bonding and lone pair regions and are in remarkable agreement with the basic concepts of the valence shell electron pair repulsion model (VSEPR) of bonding. This correspondence is striking enough that the Laplacian distribution has been proposed to provide a physical basis for the VSEPR model (Popelier, 2000 ▸; Gillespie & Robinson, 1996 ▸). Moreover, the differentiation between valence and core shell charge redistributions is straightforward based simply on the distances of the maxima of charge concentration from the nuclear position. Analysis of experimental and theoretical Laplacian distributions has also been applied to metal containing molecules and structures. For example, there are eight VSCCs for the *d* ^6^ transition metal compounds Cr(CO)_6_ (Farrugia & Evans, 2005 ▸) and iron disulfide FeS_2_ (Schmøkel *et al.*, 2014 ▸), which are arranged at the vertices of a cube where the metal–ligand vectors pass through the center of each face. Such features are referred to as ligand opposed charge concentrations and can be rationalized in the context of a simple ligand-field theory approach wherein the concentration of the metal *d* electrons reflects avoidance of the ligand charge concentrations. In addition, analysis of Laplacian distributions derived from theoretical methods suggest significant polarization of outer-core orbitals in transition metal complexes (Bader *et al.*, 1998 ▸; Batke & Eickerling, 2013 ▸).

**Figure 10:**
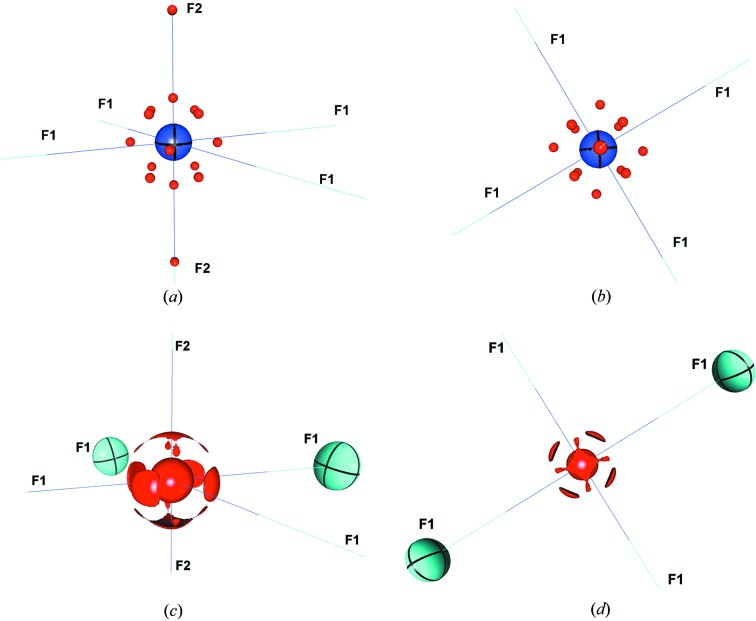
Envelope diagrams [(*c*) and (*d*)] of the negative Laplacian distribution (charge concentration) around the U atom in the UF_6_^−^ anion plotted at the −280 e Å^−5^_­_ level. The corresponding (3, +3) critical points (maxima of charge concentration) of the Laplacian distribution [(*a*) and (*b*)] are depicted as red spheres. The directions of the fluorine ligands are indicated by labels, except for the axial F2 atoms in (*b*) and (*d*), where the axial ligands are above and below the plane of the image. When depicted, thermal ellipsoids are drawn at the 99% probability level. Although these distributions were obtained from model **1c**, nearly identical distributions are obtained from model **1b**. U–cp distances are in the range 0.37–0.38 Å for the 14 nearest cps and 1.075 Å for the distant cp. The F2–cp distance is 1.001 Å.

In the present case, the valence space of uranium spans three principal quantum numbers and is further complicated by the presence of outer-core orbitals which may also deform in response to bonding. The charge concentrations observed in the preferred experimental models **1b** and **1c** are self-consistent, suggesting that asphericity of the *n* = 4 electrons is not a major contribution to the bonding picture (Fig. 10 ▸). In the case of UF_6_^−^, there are three ‘sets’ of charge concentrations surrounding the uranium atom all at a distance of ∼0.38 Å, corresponding to ‘valence shell’ charge concentrations near the radial maxima of the *n* = 5 electron density level. The first ‘set’ contains eight critical points arranged in a cube and resembles the ligand opposed charge concentrations as described above in the example of Cr(CO)_6_ (Farrugia & Evans, 2005 ▸). However, in the case of UF_6_^−^ the uranium–fluorine vectors do not pass perfectly through the faces of such a cube, but are rotated towards the metal–ligand vectors. In the second ‘set’ there are four critical points corresponding to charge concentrations which are arranged in a square in the equatorial plane. As with the first ‘set’, the metal–ligand vectors are tilted with respect to passing through the midpoint of each edge of the square. The implication of the tilting from the idealized positions as observed in this system is not immediately obvious, although we note that *d*-orbital tilting has previously been predicted to be physically meaningful in some cases and experimentally confirmed (Deutsch *et al.*, 2011 ▸). A similar set of charge concentrations was observed in a theoretical study of VF_5_ (*i.e.* three charge concentrations between equatorial ligands in this trigonal bipyramidal example; Gillespie *et al.*, 1996 ▸). The final set consists of two charge concentrations directed along the U—F2 bond vector. We also note the presence of another (3, +3) critical point (corresponding to charge concentration, although the Laplacian is positive at this critical point) along the bond nearer to the F2 atom and in the vicinity of the U—F2 bcp; interestingly, we do not find the analogous critical points along the U—F1 bond, although the Laplacian distribution along the U—F bond paths are similar (Fig. S2).

**Figure 11:**
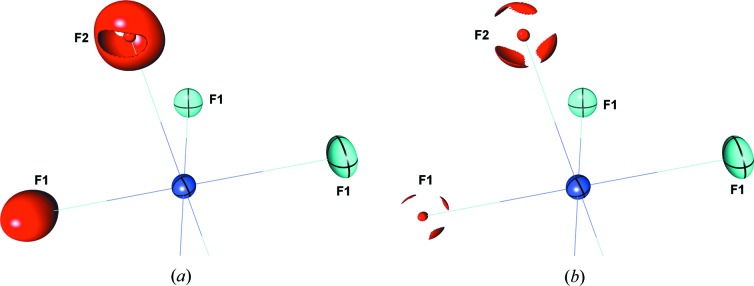
Envelope diagrams of the negative Laplacian distribution (charge concentration) around the fluorine atoms in the UF_6_^−^. The isosurfaces are plotted at (*a*) −160 and (*b*) −190 e Å^−5^_­_ levels. Thermal ellipsoids are drawn at the 99% probability level. While these distributions were obtained from model **1c**, nearly identical distributions are obtained from model **1b**.

The Laplacian distribution around the fluorine atoms also merits brief description. Neither fluorine atom demonstrates well defined features in the Laplacian distribution. Thus, until close to the maxima of charge concentration, their Laplacian distributions are nearly spherical, with weak maxima of charge concentration at a distance of ∼0.3 Å from the nucleus. These results suggest a strong ionic component and an expansion of the charge cloud with respect to a neutral F atom (valence shell radial distribution maximum ∼0.21 Å). Nevertheless, when visualized as an isosurface near the maxima of charge concentration the Laplacian distributions around both F1 and F2 resolve into three maxima (Fig. 11 ▸). In the case of F2, two maxima are directed towards uranium while the third maximum is directly behind the F2 atom opposite the U—F bond. The maxima of charge concentration around F1 are similar in that two maxima are in the direction of the uranium atom, although with some distortion compared with F2, whereas the third maximum is directed towards H4 on a nearby cation with a maximum —F1—U angle of ∼120°.

**Figure 12:**
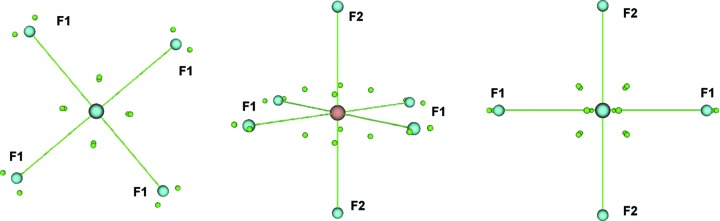
Graph depicting the (3, +3) critical points (green spheres) in the Laplacian as determined from the theoretical model of the UF_6_^−^ ion, shown in three orientations.

In contrast to the experimental result, there is only one analogous ‘set’ of critical points corresponding to charge concentrations in the Laplacian distribution around uranium for the theoretical density (Fig. 12 ▸). The theoretical CCs are arranged at the vertices of a cube at a distance of ∼0.85 Å from the uranium atom, corresponding to the radial maxima of the *n* = 6 level. In this case, the uranium–fluorine bond vectors do pass through the center of each face of the cube formed by the charge concentrations, which is expected from the point of view of ligand-field theory. Nevertheless, it is from this vantage point that disagreement between experiment and theory is most striking. It is of interest to consider whether these differences are due to the choice of RECP employed in the theoretical calculations. The small-core RECPs tend to be favored as the frozen-core ‘smooths’ features in the ‘core’ levels. Theoretical results suggest that these ‘core-wiggles’ may be important especially when trying to understand subtle features and (electronic and derived QTAIM) properties in actinide containing complexes (Odoh & Schreckenbach, 2010 ▸). As we have suggested above, it would be of interest to compare the Laplacian distribution and topological properties of simple actinide compounds at various levels of theory and with respect to the core size for RECPs utilized.

## 4. Conclusions   

Over the past decade we have sought to improve our methodology for the collection of extremely accurate, high-resolution single-crystal diffraction data at cryogenic temperatures as well as improving our data reduction techniques. These efforts have resulted in strategies for data collection, reduction and processing, and modeling, providing the opportunity to study and characterize the electron density in systems containing very heavy elements such as actinides, which were considered ‘nearly impossible’ only 30 years ago. Moreover, we have shown that experimental results are in very good agreement with those predicted by theory in the case where an augmented Hansen–Coppens scheme was used to model the aspherical electron density of the uranium atom in [PPh_4_][UF_6_]. Comparison of QTAIM properties between experimentally and theoretically derived electron densities indicates that accurate modeling requires inclusion of terms to describe deformation of the outer-core electron levels. Analogous results have previously been shown in the case of theoretical calculations. Experiment and theory both indicate that the U—F bond is of mixed character, belonging to the so-called transit region, wherein the interaction can be considered to possess both ionic and covalent character to varying degrees. While the agreement between experiment and theory is strong, there are a few noteworthy differences: first, experimentally derived electron densities suggest ∼10% more covalent character than is indicated by theory and secondly, the structure of the Laplacian distribution around the heavy atom is markedly different. In the case of experiment, the maxima of charge concentration around uranium (14 total) are in the vicinity expected for the *n* = 5 level, whereas the maxima of charge concentration for the theoretical result (8 total) correspond to the *n* = 6 level. It is of interest to consider whether or not this effect results from the RECP treatment of the core electrons for the theoretical model. Finally, differences between the axial and equatorial fluorine atoms are much more pronounced in the experimental result than suggested by theory. It is likely that crystal packing effects play some role in the differences observed in the axial and equatorial fluorine ligands. Nevertheless, we believe that these differences may provoke discussion between experimental and theoretical chemists and that such interplay will help to refine both experimental and theoretical methodologies. Moreover, these results demonstrate that meaningful results can now be obtained for experimental charge-density studies of systems containing heavy elements such as actinides.

## Supplementary Material

Crystal structure: contains datablock(s) 1b, 1c. DOI: 10.1107/S2052252519010248/lq5024sup1.cif

Structure factors: contains datablock(s) mol. DOI: 10.1107/S2052252519010248/lq50241bsup2.hkl

Structure factors: contains datablock(s) mol. DOI: 10.1107/S2052252519010248/lq50241csup3.hkl

Additional figures and tables . DOI: 10.1107/S2052252519010248/lq5024sup4.pdf

CCDC references: 1941427, 1941428, 1941428

## Figures and Tables

**Table 1:** Summary of models

Formula	[PPh_4_][UF_6_]		
Temperature	20.0 (1) K		
Spherical atom model *R* indices Δρ (max and min)	*I* > 2σ(*I*) *R*_1_ = 0.0082, *wR*_2_ all data = 0.0181, 1.291 and −1.300 e Å^−3^		
Multipole model	**1a** † 78 e FC: 1*s* to 5*d*	**1b** 60 e FC: 1*s* to 4*f*	**1c** 28 e FC: 1*s* to 3*d*
Valence pseudo-atoms	6*s*, 6*p*, 5*f*, 6*d*, 7*s*	5*s* + 5*p*, 5*d*, 6*s* + 6*p*, 5*f*, 6*d*, 7*s*	4*s* to 4*f*, 5*s* to 5*d*, 6*s* + 6*p*, 5*f*, 6*d*, 7*s*
Final *R* indices: *R*_1_ *I* > 3σ(*I*) *wR*2 all data	*R*_1_ = 0.0066, *wR*_2_ = 0.0095	*R*_1_ = 0.0066, *wR*_2_ = 0.0093	*R*_1_ = 0.0065, *wR*_2_ = 0.0084
Δρ (max and min) sin θ/λ < 1.0 Å^−1^	0.295 and −0.561 e Å^−3^	0.319 and −0.588 e Å^−3^	0.298 and −0.519 e Å^−3^

† Gianopoulos *et al.*, 2017*b* ▸.

**Table 2:** Properties of the electron density at the U—F bond critical points ρ (eÅ^−3^) is the electron density at the bcp; ∇^2^ρ (eÅ^−5^) is the Laplacian at the bcp; *d* (U–cp) (Å) is the distance from uranium to the bcp in Å; *g* (a.u.) is the electron kinetic energy density at the bcp; *v* (a.u.) is the electron potential energy density at the bcp; *h* (a.u.) is the total electron energy density at the bcp; |*v*|/*g* is the adimensional bonding regime descriptor; *h*/ρ (a.u.) is the covalence degree. The electron kinetic energy density *g* was obtained by the Abramov approximation (Abramov, 1997 ▸) and the electron potential energy density *v* according to the local Virial relationship (Bader, 1994 ▸).

	ρ (eÅ^−3^)	∇^2^ρ (eÅ^−5^)	*d* (U–cp) (Å)	*g* (a.u.)	*v* (a.u.)	*h* (a.u.)	|*v*|/*g*	*h*/ρ (a.u.)
Theory†								
U—F1	0.834	11.882	1.150	0.1703	−0.2173	−0.0470	1.276	−0.380
U—F2	0.812	11.578	1.157	0.1642	−0.2083	−0.0441	1.269	−0.367
Exp. **1a** †								
U—F1	0.930	7.676	1.157	0.1586	−0.2375	−0.0789	1.498	−0.573
U—F2	0.902	6.300	1.146	0.1439	−0.2224	−0.0785	1.546	−0.588
Exp. **1b**								
U—F1	0.868	11.016	1.169	0.1703	−0.2264	−0.0561	1.329	−0.436
U—F2	0.827	11.643	1.169	0.1673	−0.2138	−0.0465	1.278	−0.380
Exp. **1c**								
U—F1	0.881	10.545	1.162	0.1694	−0.2239	−0.0546	1.322	−0.418
U—F2	0.885	9.023	1.166	0.1595	−0.2255	−0.0659	1.413	−0.503

† Gianopoulos *et al.*, 2017*b* ▸.

**Table 3:** Maxima and FWHM for selected single-ζ radial functions, *R_l_*, for valence and outer-core electronic levels The ‘avg.’ label denotes a population based weighted-average.

Electronic level	Maximum (Å)	FWHM (Å)	FWHM range (Å)
Valence			
5*f*	0.505	0.419	0.325 to 0.744
6*d*	1.195	0.9	0.80 to 1.70
7*s*	1.685	1.15	1.175 to 2.325
Outer-core			
5*s*	0.28	0.235	0.18 to 0.415
5*p*	0.315	0.261	0.202 to 0.463
5*d*	0.36	0.302	0.23 to 0.532
5*s*—5*d* (avg.)	0.335	0.278	0.215 to 0.493
6*s*	0.63	0.472	0.425 to 0.897
6*p*	0.76	0.567	0.513 to 1.08
6*s* + 6*p* (avg.)	0.725	0.538	0.487 to 1.025
