# Supplementary material for: Charge densities in actinide compounds: strategies for data reduction and model building
Source: IUCrJ. 2019 Aug 7;6(Pt 5):895–908. doi: 10.1107/S2052252519010248 (PMC6760433; doi:10.1107/S2052252519010248)
Supplement: Supplementary file 4 [file m-06-00895-sup4.pdf]

IUCrJ

Volume 6 (2019)

Supporting information for article:

Charge densities in actinide compounds: strategies for data reduction and model building

Christopher G. Gianopoulos, Vladimir V. Zhurov and A. Alan Pinkerton

Table of Contents

Figure S1. Uranium atom radial probability distributions and radial density for selected (spherical) ρ(n,l). 3

Figure S2. $\nabla^2\rho$ plotted along the U–F bond paths 4

Table S1. Intermolecular interactions characterized by bond paths involving the F 5

Figure S3. Normal probability plots for the IAM model and models **1a**, **1b** and **1c**. 6

Figure S4. Averaged scale factor plots binned by 0.5 Å^{-1} increments for all models. 7

Figure S5. Full scale factor plots for the IAM model and models **1a**, **1b** and **1c**. 8

Figure S6. Residual density maps for the IAM model and models **1a**, **1b** and **1c**. 9

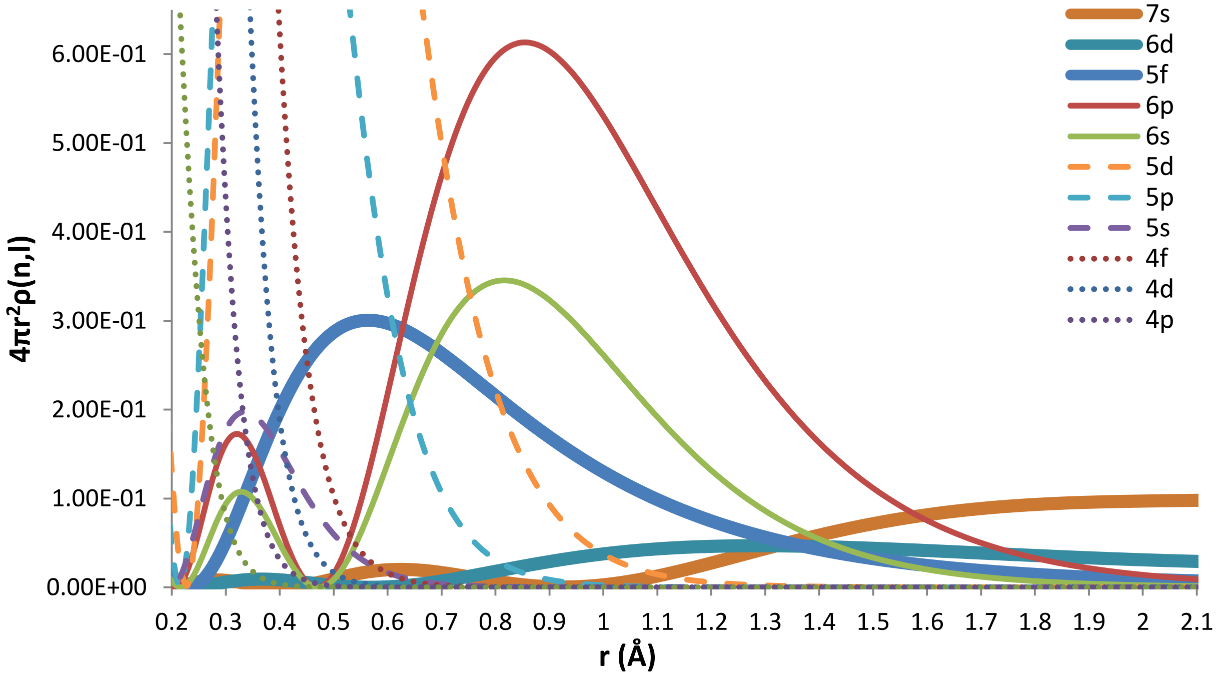

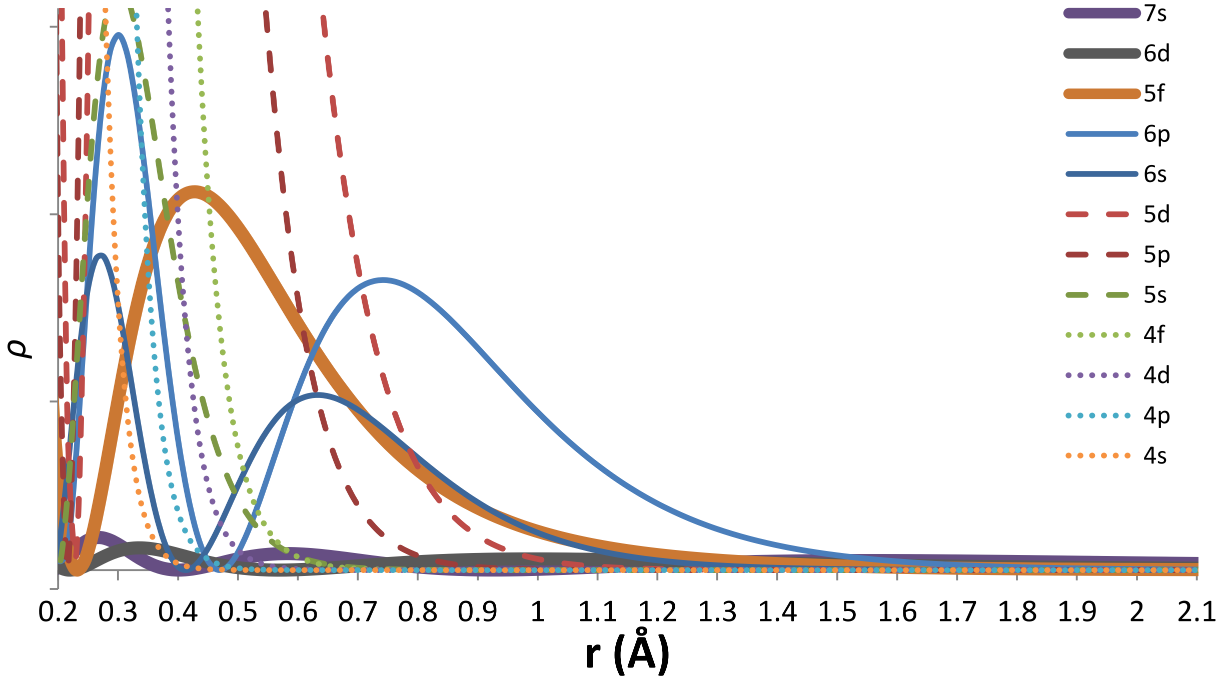

Figure S1. Uranium atom (above) radial probability distributions (unnormalized) and radial density (below) for selected (spherical) ρ(n,l) against distance (Å) plotted to the U-F bond distance in $UF6^-$. Valence distributions in bold, 6s and 6p thin lines, 5s-5d dashed lines, 4s-4f dotted lines.

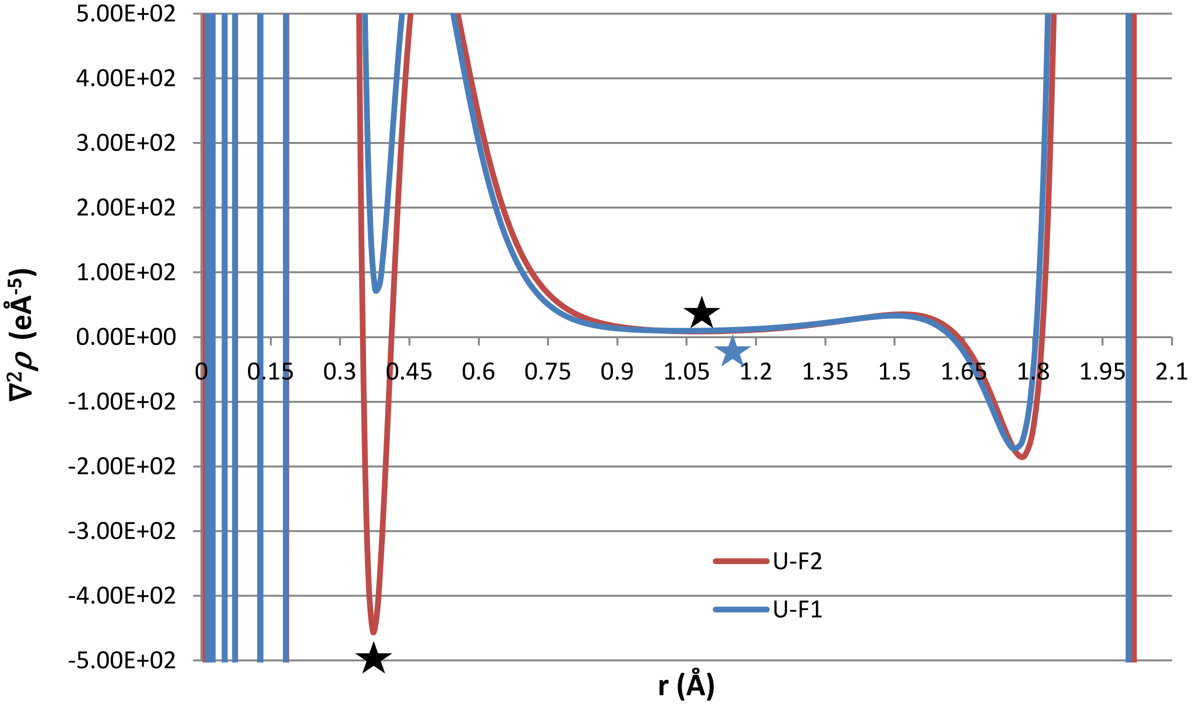

Figure S2. $\nabla^2\rho$ plotted along the U–F bond paths. Approximate locations of the (3,+3) critical points in the Laplacian along the U–F2 bond are denoted by black stars. The approximate location(s) of the U–F1 and U–F2 bond critical points in ρ is denoted by a blue star.

Table S1. Intermolecular interactions characterized by bond paths involving the F atoms for model **1b** (above) and **1c** (below). Symmetry operators: a = *y*, 1 – *x*, 1 – *z*; b = *y*, 1 – *x*, –*z*; c = 0.5 – *x*, 1.5 – *y*, –0.5 + *z*; d = 1.5 – *y*, 0.5 + *x*, 0.5 – *z*.

**1b**								
Atom1	Atom2	Rij (Å)	ρ (eÅ^{-3})	$\nabla^2\rho$ (eÅ^{-5})	*g* (au)	*v* (au)	*h* (au)	De (kJ/mol)
F2	F2a	3.136	0.024	0.41	0.0031	-0.0019	0.0012	2.5
F2	H5	2.406	0.034	0.79	0.0059	-0.0036	0.0023	4.7
F2	H5a	2.589	0.053	0.68	0.0056	-0.0041	0.0015	5.4
F1	C3d	3.158	0.038	0.49	0.0039	-0.0027	0.0012	3.6
F1	H2c	2.601	0.057	0.77	0.0064	-0.0047	0.0017	6.2
F1	H4a	2.610	0.048	0.61	0.0050	-0.0036	0.0014	4.8
F1	H6b	2.520	0.045	0.70	0.0056	-0.0038	0.0018	5.0

**1c**								
Atom1	Atom2	Rij (Å)	ρ (eÅ^{-3})	$\nabla^2\rho$ (eÅ^{-5})	*g* (au)	*v* (au)	*h* (au)	De (kJ/mol)
F2	F2a	3.136	0.025	0.41	0.0031	-0.0019	0.0012	2.5
F2	H5	2.406	0.035	0.78	0.0058	-0.0036	0.0023	4.7
F2	H5a	2.589	0.054	0.68	0.0056	-0.0042	0.0014	5.5
F1	C3d	3.158	0.041	0.50	0.0040	-0.0029	0.0011	3.8
F1	H2c	2.595	0.060	0.78	0.0065	-0.0049	0.0016	6.4
F1	H4a	2.619	0.049	0.67	0.0054	-0.0039	0.0015	5.1
F1	H6b	2.515	0.047	0.71	0.0056	-0.0039	0.0017	5.1

Rij, internuclear distance in angstroms; ρ (eÅ^{-3}), electron density at the bcp; $\nabla^2\rho$ (eÅ^{-5}), Laplacian at the bcp; d (U–cp) (Å), distance from uranium to the bcp in angstroms; *g* (*au*), electron kinetic energy density at the bcp in atomic units; *v* (*au*), electron potential energy density at the bcp in atomic units; *h* (*au*), total electron energy density at the bcp; De (kJ/mol), estimated dissociation energy*.

*Espinosa, E.; Lecomte, C.; Molins, E., Experimental electron density overlapping in hydrogen bonds: topology vs. energetics. *Chemical Physics Letters* **1999,** *300* (5), 745-748.

Espinosa, E.; Molins, E.; Lecomte, C., Hydrogen bond strengths revealed by topological analyses of experimentally observed electron densities. *Chemical Physics Letters* **1998,** *285* (3), 170-173.

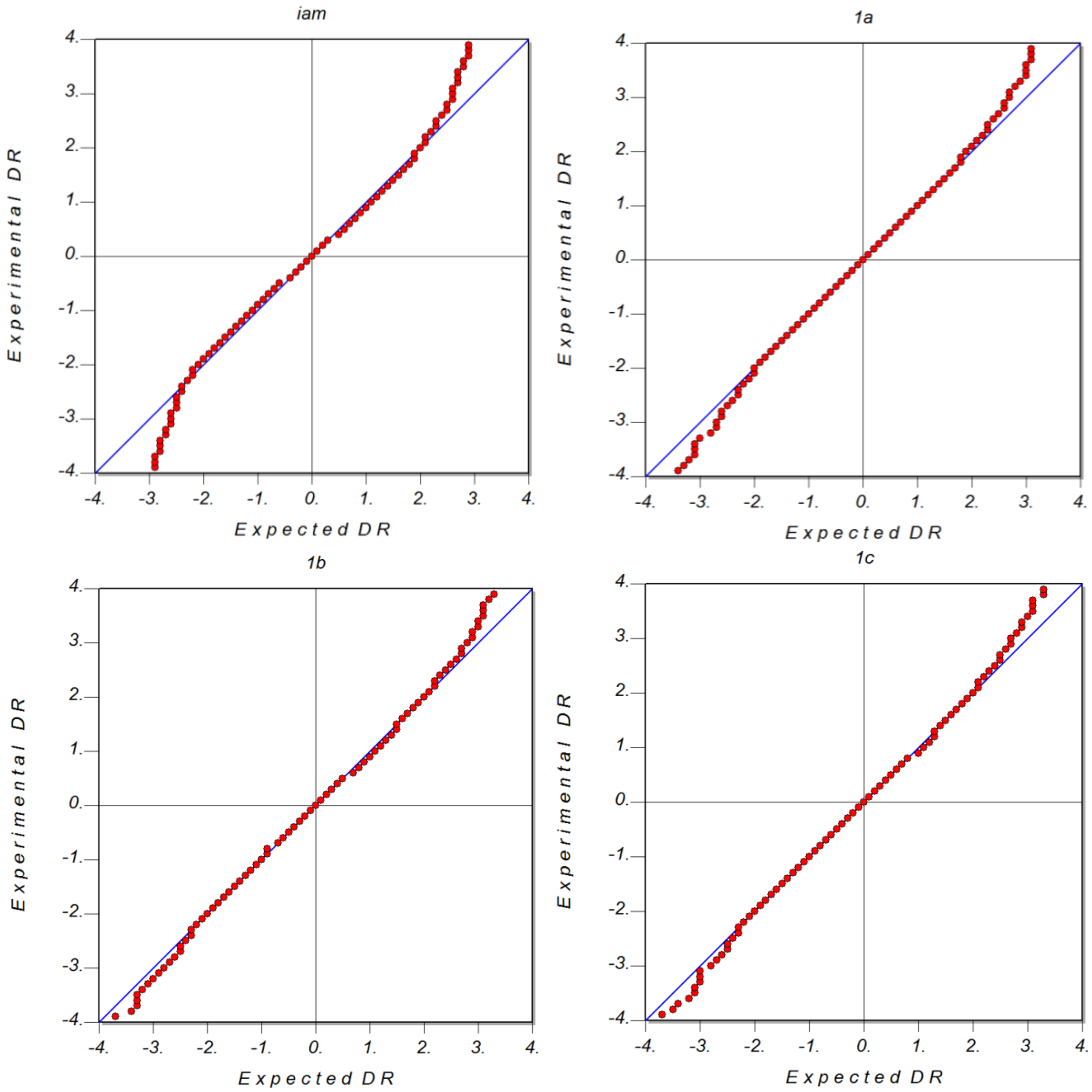

Figure S3. Normal probability plots for the IAM model and models **1a**, **1b** and **1c**.

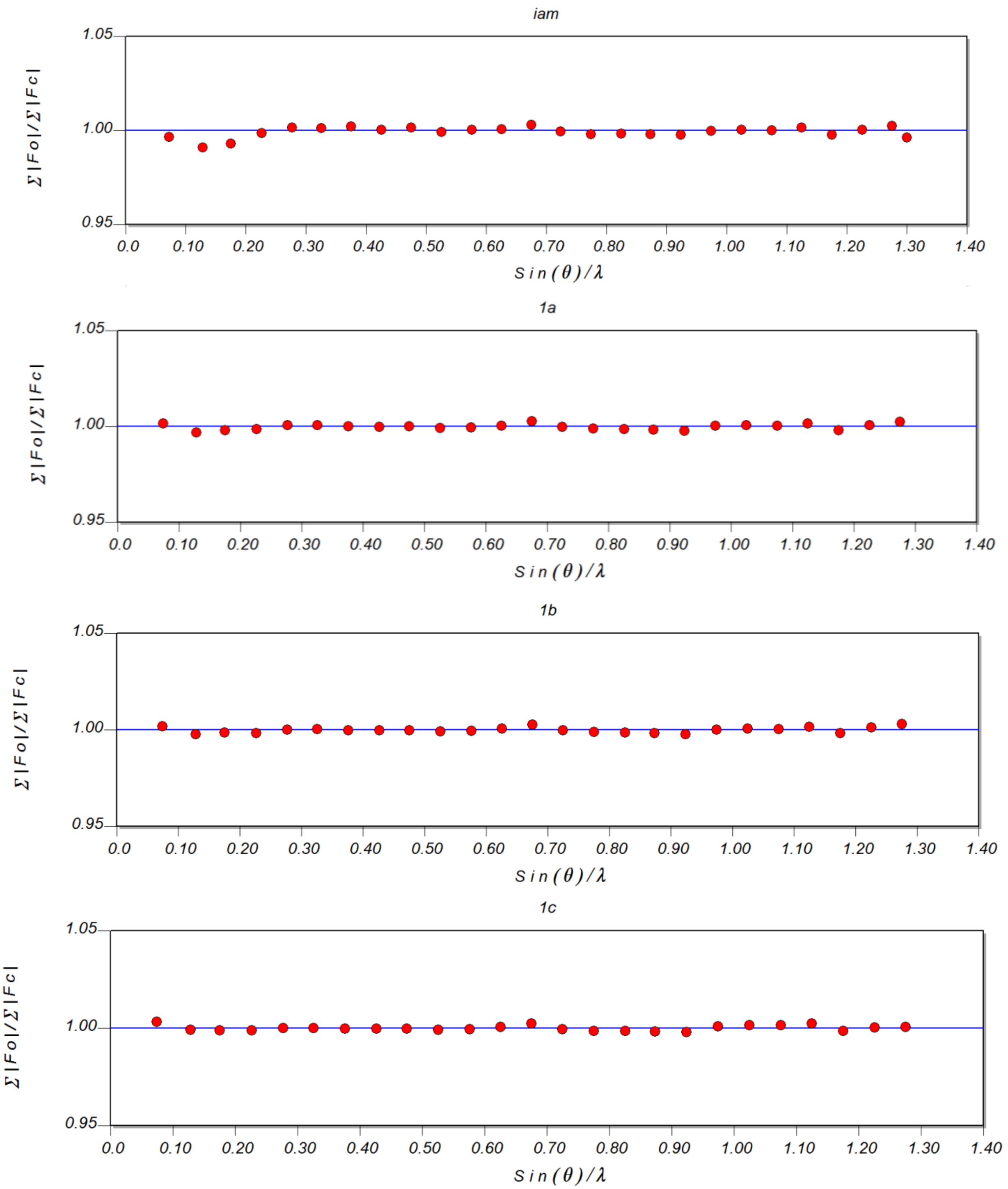

Figure S4. Averaged scale factor plots binned by 0.5 Å^{-1} increments for the IAM model and models **1a**, **1b** and **1c**.

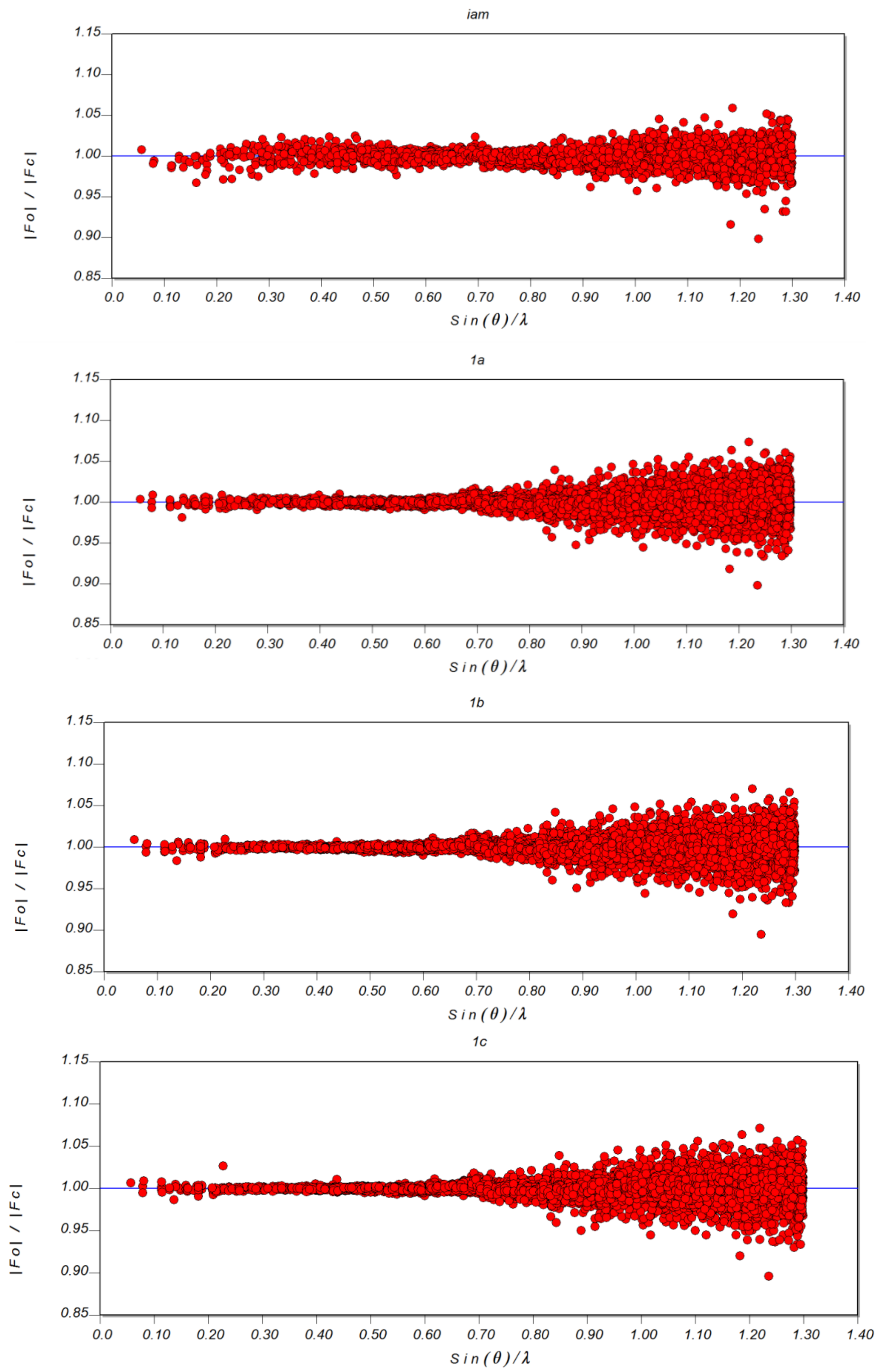

Figure S5. Full scale factor plots for the IAM model and models **1a**, **1b** and **1c**.

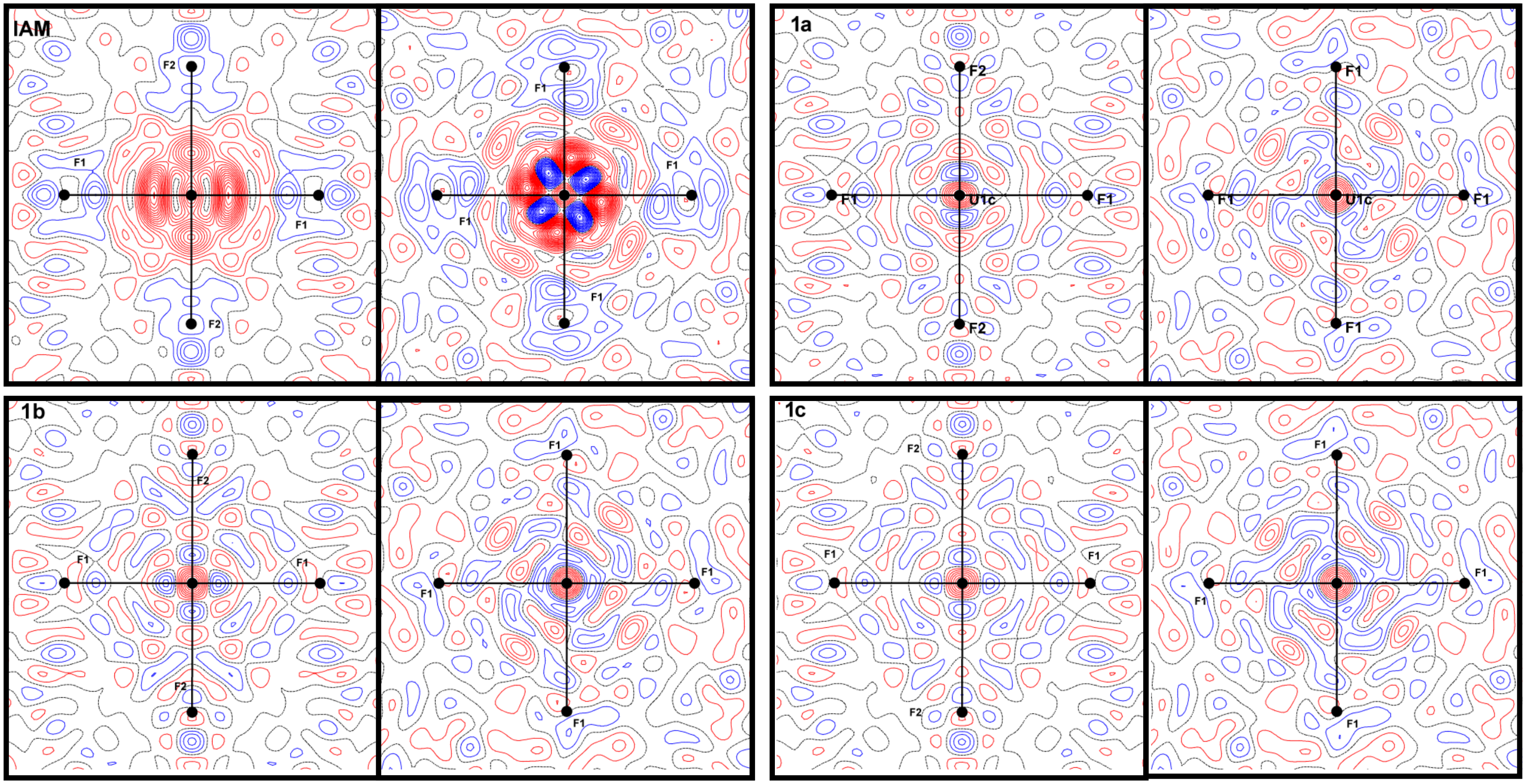

Figure S6. Residual density plots ((sin θ /λ)$_{max}$ = 1 Å^{-1}; contours are at ± 0.1 eA^{-3}; Fo > Fc is in blue and Fo < Fc in red). Two panes provided, U–F1–F2 and U–F1–F1 for each of four models (IAM, top left; **1a**, top right; **1b**, bottom left; **1c**, bottom right).
